# Analysis of the pathogenic potential of nosocomial *Pseudomonas putida* strains

**DOI:** 10.3389/fmicb.2015.00871

**Published:** 2015-08-25

**Authors:** Matilde Fernández, Mario Porcel, Jesús de la Torre, M. A. Molina-Henares, Abdelali Daddaoua, María A. Llamas, Amalia Roca, Victor Carriel, Ingrid Garzón, Juan L. Ramos, Miguel Alaminos, Estrella Duque

**Affiliations:** ^1^Department of Environmental Protection, Estación Experimental del Zaidín, Consejo Superior de Investigaciones CientíficasGranada, Spain; ^2^Bio-Iliberis R&DGranada, Spain; ^3^Unit of Integrated Plant Protection, Department of Plant Protection Biology, Swedish University of Agricultural SciencesAlnarp, Sweden; ^4^Abengoa ResearchSevilla, Spain; ^5^Department of Histology (Tissue Engineering Group), Faculty of Medicine, University of Granada and Instituto de Investigación Biosanitaria IbsGranada, Spain

**Keywords:** *Pseudomonas*, opportunistic pathogen, laminin, hospitalary strains

## Abstract

*Pseudomonas putida* strains are ubiquitous in soil and water but have also been reported as opportunistic human pathogens capable of causing nosocomial infections. In this study we describe the multilocus sequence typing of four *P. putida* strains (HB13667, HB8234, HB4184, and HB3267) isolated from in-patients at the Besançon Hospital (France). The four isolates (in particular HB3267) were resistant to a number of antibiotics. The pathogenicity and virulence potential of the strains was tested *ex vivo* and *in vivo* using different biological models: human tissue culture, mammalian tissues, and insect larvae. Our results showed a significant variability in the ability of the four strains to damage the host; HB13667 did not exhibit any pathogenic traits, HB4184 caused damage only *ex vivo* in human tissue cultures, and HB8234 had a deleterious effect in tissue culture and *in vivo* on rat skin, but not in insect larvae. Interestingly, strain HB3267 caused damage in all the model systems studied. The putative evolution of these strains in medical environments is discussed.

## Introduction

Bacteria of the genus *Pseudomonas* are ubiquitous inhabitants of soil, water, plant surfaces, and animal tissues; this phenomenon is due to their ability to establish in different niches (Walker et al., 2004; Perkins et al., 2010), and is driven mainly by their high metabolic versatility (Oberhardt et al., 2008). *Pseudomonas aeruginosa* is an opportunistic pathogen of humans, animals, and plants (Green et al., 1974; Cao et al., 2001; Attila et al., 2008; Sheng et al., 2012). They are also a frequent cause of hospital-acquired infections, including ventilator associated pneumonia (Chastre and Fagon, 2002) and catheter infections in immuno-compromised patients, in addition they are the main cause of mortality in cystic fibrosis (CF) patients (Høiby, 2002; McGuigan and Callaghan, 2015). *P. aeruginosa* strains are often highly resistant to antibiotic treatment and are therefore very difficult to eradicate once established in the host (Høiby et al., 2010; Bodro et al., 2015).

Strains of the species *Pseudomonas putida* are frequent rhizosphere and freshwater inhabitants and exhibit an amazing ability to metabolize a wide range of biogenic and xenobiotic compounds (Fernández et al., 2012; Udaondo et al., 2013). Several strains of this species have been isolated from patients who have acquired infections in hospital environments. Infections caused by *P. putida* are rare and are mostly reported in immuno-compromised individuals, such as those with neutropenia, newborns, and cancer patients (Lombardi et al., 2002; Kim et al., 2012; Erol et al., 2014). Most isolates exhibit resistance to certain antibiotics through the presence of plasmids bearing the genes that encode antibiotic resistance factors (Molina et al., 2014), which can be transferred to other microorganisms in hospital environments (Martino et al., 1996; Yoshino et al., 2011).

Despite the fact that *P. putida* may cause healthcare-related infections, clinical data on *P. putida* infections is scarce; this likely due to the rarity, relatively lower virulence, and higher antimicrobial susceptibility of *P. putida* compared to *P. aeruginosa* (Blazevic et al., 1973; Carpenter et al., 2008; Yoshino et al., 2011). The recent emergence of multi-drug-resistant (MDR) and carbapenem-resistant *P. putida* has become a cause for concern (Almuzara et al., 2007; Bennett et al., 2009); although this has not been extensively investigated (Kim et al., 2012).

Our group recently reported the antibiotic resistance determinants of HB3267, a *P. putida* strain isolated at the Jean Minjoz Hospital (Besançon, France; Molina et al., 2014). In fact, HB3267 is one of several *P. putida* strains that were isolated from inpatients at this hospital and multilocus sequence typing (MLST) let us distinguish at least four different types: HB13667, HB8234, HB4184, and HB3267. In this study we evaluated the potential risk of infection and pathogenicity of the four *P. putida* strains isolated from the clinical samples. Analysis included antibiotic resistance profiles and biofilm formation. Pathogenicity and virulence were tested using different biological models (human tissue and insect cultures); in all model systems, one of these strains, HB3267 (present in several clinical samples from Jean Minjoz Hospital), showed the ability to cause damage to the host, and exhibited resistance to a large number of antibiotics. The other isolates exhibited a profile of resistant to a number of antibiotics of clinical use. HB8234 had a deleterious effect on tissue cultures and *in vivo* mammalian cultures, but not on insect larvae, while HB4184 only caused damage *ex vivo* in human tissue culture. One isolate, HB13667 did not cause harm to the host in the models tested. Our data indicate that although *P. putida* may be associated with clinical disease, there is in fact a great deal of variability amongst strains in regard to drug resistance and pathogenicity.

## Results

### Clinical Strains *P. putida* Species Confirmation

Eight clinical isolates pre-identified as *P. putida* at the Jean Minjoz Hospital (Besançon, France; **Table 1**) were subjected to a set of tests to verify that they belong to this species. The tests included: (a) Sequence analysis of DNA 16S fragment >500 bp, according to Janda and Abbott (2007); (b) biochemical identification using API 20E tests (BioMerieux, Inc.), and (c) PCR amplification of species-specific repetitive extragenic palindromic sequences for *P. putida* (REPc profile), which enables us to identify exclusively *P. putida* strains and even discriminate among different strains (Aranda-Olmedo et al., 2002).

**Table 1:** Bacterial strains used in this study.

Species	Strain	Source	Reference
*Pseudomonas putida*			
**Hospital Besançon isolates**			
	HB13667	Bacteraemia/co-isolated with *Enterobacter cloacae*	This study
	HB8234	Bacteraemia	Molina et al. (2013)^∗^
	HB3304	Cystic fibrosis	This study
	HB4184	Cystic fibrosis	This study
	HB4477	Urinary infection	This study
	HB4557	Urinary infection	This study
	HB3536	Faeces	This study
	HB3267	Deceased in-patient	Molina et al. (2014)
**Environmental strain**	KT2440	Lab. Collection	
*Pseudomonas aeruginosa*	PAO1	Lab. Collection	
*Escherichia coli*	DH5α	Lab. Collection	

*^∗^Strain was firstly denoted as H8234.*

All the rRNA16S gene sequences analyzed exhibited similarities of at least 99% with *P. putida* strains available on database. In addition, the eight strains were positively identified as *P. putida* by the API tests; three different REPc profiles were detected, which suggested the existence of different *P. putida* strains. Taken together these results unequivocally confirmed that the hospital isolates belonged to the species *P. putida*.

### Multilocus Sequence Typing

With the aim of determining how similar the clinical isolates were at the genetic level and the phylogenetic relationship among them and other *P. putida* strains we performed MLST using the complete sequence of five highly conserved genes: *ropD*, *gyrB*, *edd*, *recA*, and *trpF* (which in total sum more than 7 kb). When the resulting sequences were compared using BLAST, in all cases maximum likelihood similarities were found with respective to homologous genes in *P. putida* soil isolates, however, the similarities did not reach 100%, indicating that these clinical strains were different from soil isolates.

**FIGURE 1:**
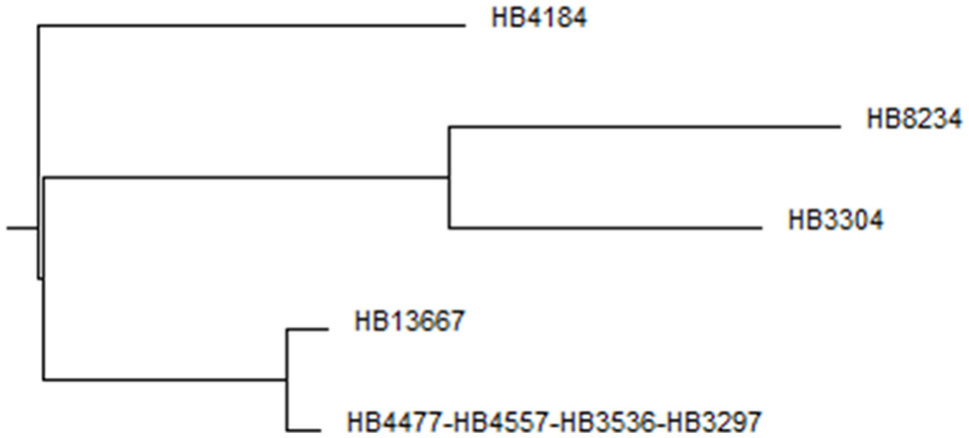
**Phylogenetic tree.** Multi-way DNA alignment of the concatenated housekeeping gene sequences.

After analyzing the sequences by Local Comparison using the Clone Manager Suite 7 program, the results allowed us to establish five allelic types and a three-branch phylogenetic tree (**Figure 1**). Four of the isolates belonged to the same allelic type: HB4477, HB4557, HB3536, and HB3267; all of them also harbored a 80 kb megaplasmid and exhibited a similar antibiotic resistance profile (see below), this data suggests that they represent different isolates of the same strain, we therefore randomly chose HB3267 as a representative of this group. Although HB13667 was phylogenetically close to the HB3267 group, the absence of the megaplasmid in this strain and a different antibiogram (see below) led us to consider it independently. HB4184, isolated from a CF patient, was dissimilar enough to be considered a different subgroup. HB8234 and HB3304 were found to be phylogenetically very similar when compared to the other clones; HB3304 was omitted from further analysis because we had already chosen a CF patient isolate. In summary and based on the above set of results, we chose HB13667, HB8234, HB4184, and HB3267 as representatives of the respective subgroups for further analysis.

### Study of Potential Pathogenic Traits in *P. putida* Clinical Strains

#### Antibiotic Resistance Profile

Antibiotic resistance tests were performed in solid medium using commercial antibiotic discs; a total of 31 antimicrobials were assayed. Results (Supplementary Table S1) showed two distinct antibiotic resistance profiles: (a) clones resistant to a high number (around 84%) of the antibiotics tested, this was the HB3267 strain (the same profile was seen with HB4477, HB4557, and HB3536). HB3267 (as well as HB4477, HB4557, and HB3536) harbor an 80 Kb megaplasmid, pPC9, previously shown to be required for antibiotic resistance (Molina et al., 2014) and (b) the rest of the clones were resistant to half of the antibiotics tested; no plasmid was found in isolates belonging to this second group of clones.

#### Analysis of the Ability to form Biofilms

**FIGURE 2:**
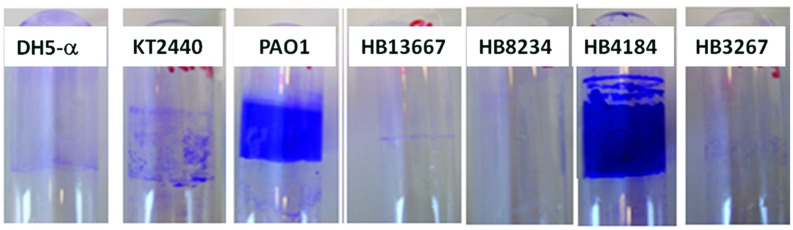
**Biofilm formation.** Bacterial biofilms stained with crystal violet.

Many pathogenic bacteria are highly resistant to antibiotic treatment, and one important antibiotic resistance mechanism is the formation of biofilms (Høiby et al., 2001; Tseng et al., 2013). To assess the capacity to form biofilms, the hospital isolates of *P. putida* were cultured in 24-well plates for 6 h, followed by a quantification of biofilm formation (see Materials and Methods). The relative amount of biofilm produced by the four strains is shown in **Figure 2**. HB4184 produced very dense biofilms with the surfaces coverage being higher than that of the positive control, *P. aeruginosa* PAO1. The other three strains (HB13667, HB8234, and HB3267) showed a limited ability to form biofilms.

### *Ex Vivo* Effects of *P. putida* Strains on Human Skin Cultures

#### Determination of Cell Viability by DNA and LDH Quantification

The effect of *P. putida* hospital isolates on human skin cell cultures was determined by quantifying the amount of DNA and lactate dehydrogenase (LDH) released by damaged cells. This analysis revealed that all strains were able to increase the amount of DNA released from skin cells to the culture medium; the results corresponding to HB8234, HB4184, and HB3267 were significantly higher than negative controls (*P* < 0.05; **Table 2**). Interestingly, the amount of DNA released from cell cultures incubated with the HB3267 strain was very similar to the positive control tissues which were treated with triton X-100 (**Table 2**).

**Table 2:** Analysis of cell viability of human skin samples incubated in the presence of the different *P. putida* strains.

	HB13667	HB8234	HB4184	HB3267	Triton-X^∗^	PBS^∗∗^
Percentage of DNA released	16.25	27.12	15.02	95.64	100.00	0.00
Percentage of LDH released	12.30	24.71	17.52	32.52	100.00	0.00

*Cytotoxicity was determined by DNA and LDH release after 24 h of infection. ^∗^Triton X-100 detergent was used as a positive control (100% cell death). ^∗∗^Bacteria-free PBS was used as a negative control (100% cell survival).*

#### Histological Analysis of Human Skin Samples Incubated with *P. putida* Strains

**FIGURE 3:**
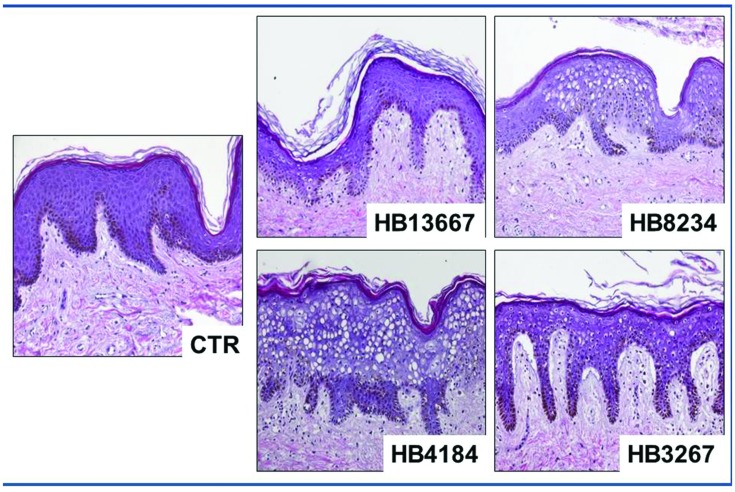
**Histological analysis of human skin using light microscopy, samples incubated with the different *Pseudomonas putida* strains (HB13667, HB8234, HB4184, and HB3267) and control non-inoculated skin (CTR).** All samples were stained with hematoxylin and eosin.

The histological analysis of human skin fragments incubated in the presence of the different bacteria strains showed that all strains were able to alter the structure of both the epithelium and the stromal layer of the human skin, this was particularly notable for strains HB8234, HB4184, and HB3267. In all cases, we found vacuolization and nuclear degeneration of epithelial cells along with dermal disorganization when compared to control human skin (**Figure 3**).

#### Immunohistochemistry and Immunofluorescence for Epithelial Cell Markers

**FIGURE 4:**
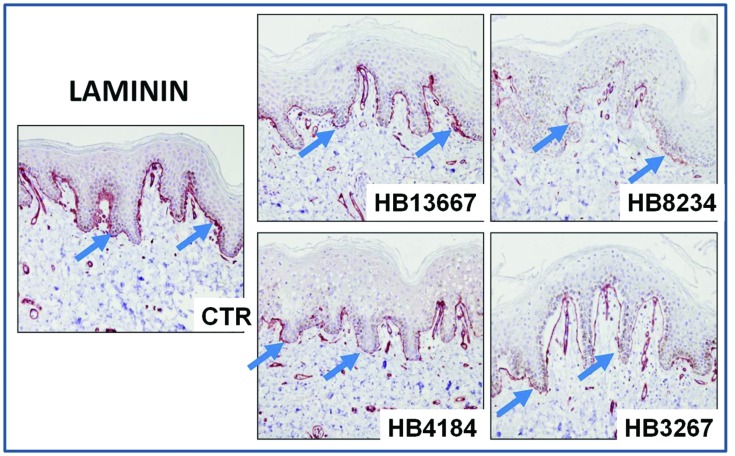
**Analysis of the integrity of the basement membrane of human skin samples incubated with the different *P. putida* strains (HB13667, HB8234, HB4184, and HB3267) and control non-inoculated skin (CTR) using laminin immunohistochemistry.** Positive signal is labelled in brown and highlighted with arrows.

In order to determine if the strains were capable of disrupting the epithelial layers, we tested important structural proteins of the skin using immunohistochemistry and immunofluorescence. Immunohistochemical detection of laminin, which is one of the most important components of the basement membrane of the epithelium, showed significant alterations of the dermal-epidermal junction of tissues incubated with the different strains, as shown in **Figure 4**. The most damaged laminin was found for HB3267, which showed a clear separation of the epithelial and stromal layers.

**FIGURE 5:**
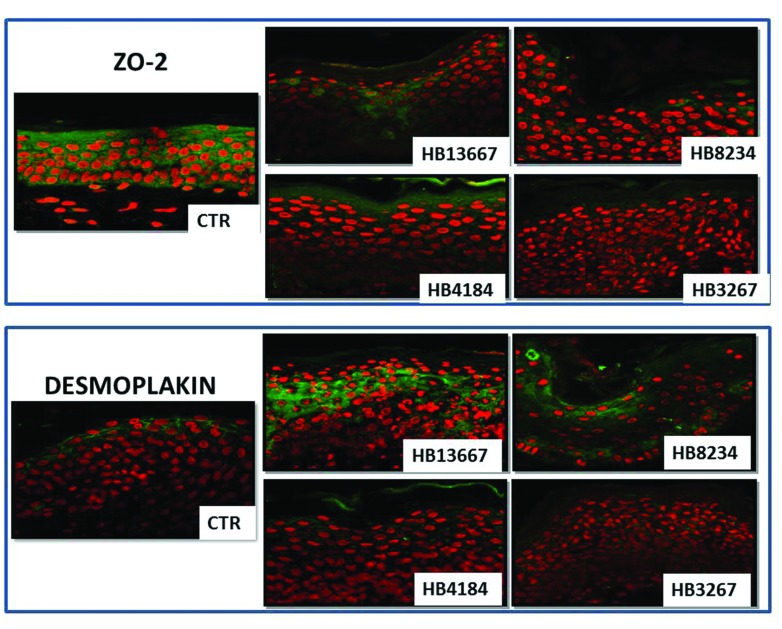
**Analysis of the integrity of epithelial cell-cell junctions as determined by immunohistofluorescence for ZO-2 (**upper**) and desmoplakin (**lower**) in human skin samples incubated with the different *P. putida* strains (HB13667, HB8234, HB4184, and HB3267) and control non-inoculated skin (CTR).** Positive signal is labelled in green and cell nuclei have been counterstained in red.

When two specific proteins with a key role in cell-cell attachment (ZO-2 and desmoplakin) were analyzed by immunofluorescence, we found that the signal intensity of both components of the cell-cell junction complex were reduced after exposer to the different *P. putida* strains (**Figure 5**). Incubation of the human skin tissues in the presence of HB13667, HB8234, and HB3267 significantly reduced the expression of ZO-2, a key component of the tight junctions which maintain the barrier function of the human skin. We also found that desmoplakin, a protein with an important role in cell-cell attachment via adherent junctions, was altered in skin incubated in all strains; again this was especially noticeable in the presence of HB3267.

#### Immunohistochemistry for Immune-System Cell Markers

To determine the immune response initiated by the presence of the different strains *in vitro*, we analyzed several immune-related markers using immunohistochemistry in controls and skin incubated with the different bacterial strains. The results of this analysis showed increased expression of TNF-positive cells in the dermis of human skin incubated in HB4184 and HB3267. At the same time, we also found that samples in which the HB3267 strain had been inoculated had higher number of cells which showed positive expression of IL-1A, IL-6, and IF in the dermal layer (**Table 3**).

**Table 3:** Percentage of dermal cells showing positive expression of tumor necrosis factor (TNF), interleukin 1A (IL-1A), interleukin 6 (IL-6), and interferon (IF) in human skin samples inoculated with the different *P. putida* strains (HB13667, HB8234, HB4184, and HB3267) and control non-inoculated skin as determined by immunohistochemistry.

Marker	HB13667	HB8234	HB4184	HB3267	Control
TNF	15–20%	5–10%	10–15%	30–40%	5–10%
IL-1A	5–10%	5–10%	5–10%	5–10%	5–10%
IL-6	20–30%	20–30%	20–30%	30–40%	20–30%
IF	5–10%	<5%	5–10%	10–20%	20–30%

### *In Vivo* Effects of the *P. putida* Strains on a Mammalian Model

**FIGURE 6:**
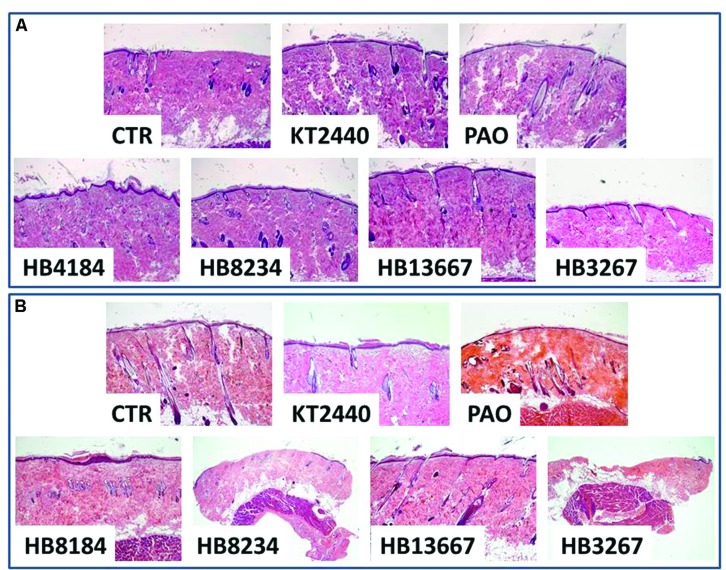
***In vivo* analysis of the effects of the different *P. putida* strains (HB13667, HB8234, HB4184, and HB3267) and controls on intact rat skin (A) and freeze-injured rat skin (B)**. All samples were fixed in formalin, stained with hematoxylin-eosin and analyzed using a light microscope. CTR: control non-inoculated skin. KT-2440: skin inoculated with non-pathogenic KT-2440 strains (negative control). PAO1: skin inoculated with *P. aeruginosa* (positive control).

The effects of the *P. putida* strains were evaluated *in vivo* on the skin of Wistar laboratory rats. Clinical analysis of the skin of animals in which the different strains had been inoculated did not reveal any macroscopically detectable skin damage in any of the groups. However, histological analysis demonstrated the existance of structural differences between the samples. In accordance with *P. putida* being an opportunistic pathogen, none of the animals with intact skin displayed any histological alteration, however, the inoculation of certain bacteria on the rat skin previously damaged with liquid nitrogen was associated with different levels of tissue organisation (**Figure 6**). Specifically, the skin epidermis was seriously damaged in samples treated with HB3267 and, at lesser extent, HB8234. In both cases, the epithelial damage was significantly higher than that observed for control skin inoculated with the positive control -*P. aeruginosa*- and the negative control -KT2440- in the same animals with a liquid nitrogen freeze injury. The same trend was found in the dermis, although the damage was less intense than in the epidermis, with the exception of skin treated with HB3267, where a high percentage of connective tissue was damaged and lost after 24 h of incubation with this strain.

### Effects of *P. putida* Clinical Strains on an Insect Model: *Chrysoperla carnea*

**FIGURE 7:**
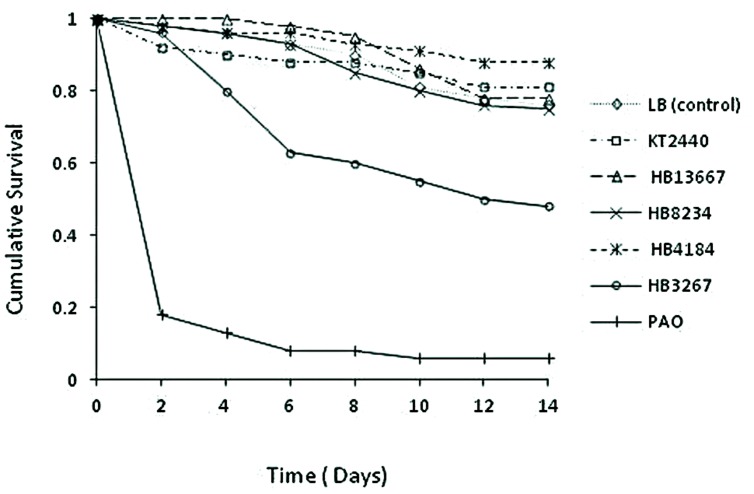
**Cumulative survival of *Chrysoperla carnea* larvae treated with different *P. putida* clinical strains**.

*Chrysoperla carnea* larvae were put in contact with bacterial strains PAO1, KT2440, and clinical isolates HB13667, HB8234, HB4184, and HB3267. *P. aeruginosa* PAO1, used as positive control, gave a 94% mortality rate, with a 82% of mortality within 2 days of inoculation (89.3% of the total of deaths; **Figure 7**). As expected, the environmental strain KT2440 had a minimal effect on larvae mortality, with <20% mortality after 12 days incubation, a similar effect was seen with the HB13667, HB8234, and HB4184 clinical isolates. In contrast, only 48% of the larvae exposed to the strain HB3267 survived; an importantly a high rate of mortality was observed between days 3 and 5 following inoculation with 64.7% of the total deaths occurring in this period (**Figure 7**). The Cox model revealed statistically significant differences between the risks of mortality produced by PAO1 and HB3267, and between these two and the other *P. putida* tested (**Table 4**). Therefore, only PAO1 and HB3267 showed an increase mortality risk than the control. The effect of the frailty term was not significant in the model, indicating no difference between experimental runs over time.

**Table 4:** Hazard ratios of *C. carnea* larvae in contact with *P. putida* clinical strains. Cox model analysis.

Factor	Hazard ratio	95% Confidence interval	Wald chi-square	df	*P*
Treatment*^a^*			244.5	6	<0.001
LB control (A)	1.00				
KT2440 (B)	0.80	0.36–1.77	0.30	1	0.580
HB13667 (C)	0.97	0.46–2.03	0.01	1	0.930
HB8234 (D)	1.10	0.53–2.28	0.07	1	0.800
HB4184 (E)	0.54	0.23–1.29	1.90	1	0.170
HB3267 (F)	2.87	1.52–5.41	10.68	1	0.001
PAO1 (G)	21.25	11.55–39.10	96.56	1	<0.001
Block*^b^*			6.12	3.03	0.110

*^a^Pairwise comparisons of treatments: B – C, P = 0.640; B – D, P = 0.430; B – E, P = 0.400; B – F, P < 0.001; B – G, P < 0.001; C – D, P = 0.730; C – E, P = 0.190; C – F, P < 0.001; C – G, P < 0.001; D – E, P = 0.110; D – F, P = 0.002; D – G, P < 0.001; E – F, P < 0.001; E – G, P < 0.001; F – G, P < 0.001.*

*^b^Variance of random effect = 0.0415.*

## Discussion

Infections caused by multidrug-resistant Gram-negative bacteria have become a growing challenge in hospital environments; in fact these microorganisms cause a higher number of nosocomial infections than resistant Gram-positive bacteria (methicillin-resistant *Staphylococcus aureus* or vancomycin-resistant *Enterococcus* species; Leneveu-Jenvrin et al., 2013; Ott et al., 2013). *P. putida* strains are seldom isolated from clinical samples, suggesting that they survive in the hospital setting and occasionally cause nosocomial infections in severely ill or immuno-compromised patients (Carpenter et al., 2008; Treviño et al., 2010; Liu et al., 2014). Nevertheless, there are no published studies focused on evaluating the pathogenicity of *P. putida* clinical strains and the differences between clinical and environmental *P. putida* isolates have not been established. This study aimed to evaluate the pathogenic potential of *P. putida* isolates from in-patients at Besançon Hospital using different biological models.

To explore putative phenotypic differences among environmental and clinical isolates, the well-known environmental strain *P. putida* KT2240 was included as a control in this study. KT2440 is a biologically safe microorganism as declared by the Recombinant DNA Advisory Committee and it is considered a model microorganism for a variety of process (Fernández et al., 2012). Indeed, we confirm here that KT2440 did not damage host cells, in either *ex vivo* or *in vivo* experiments, where its presence was indistinguishable from bacteria-free negative controls.

### Variation in the Pathogenic Potential of *P. putida* Clinical Strains

Taken together, data from this study, have revealed the variability in the pathogenic traits exhibited between the *P. putida* strains tested; this variability is consistent with the number of phylogenic types found.

### HB3267

Among the clinical strains tested the highest pathogenic potential was associated with HB3267, which exhibited pathogenic traits in almost all of the assays: it had a cytotoxic effect on human skin cells, it caused damage to the epithelial layer, and it provoked an immune-system response. When inoculated into wounds on rats HB3267 caused severe damage to the epidermis and the underlying connective tissue. Finally, it showed a deleterious effect on the survival of *C. carnea* larvae.

Molina et al. (2014) showed that this strain was phylogenetically closed to the nicotine degrader *P. putida* S16 (Wang et al., 2007) but phylogenetically distant from *P. aeruginosa* strains. In our *in vivo* experiments the pathogenic nature of HB3267 showed low coincidence with those found for PAO1: HB3267 caused more serious damage on the injured skin of rats than PAO1, but it caused a lower mortality of *C. carnea* larvae which also occurred at different stages of insect development. This suggests the potential existence of different mechanisms of virulence in both species.

HB3267 (or variants of this strain) were isolated from four different patients at the Besançon Hospital, the rest of strains tested were isolated only once. This finding could be linked to its innate resistance to most of the antibiotics tested, since antibiotic resistance is considered a key factor in nosocomial prevalence (Bereket et al., 2012). Previous studies from our research group showed that HB3267 antibiotic resistance is encoded both on the chromosome and the pPC9 plasmid (Molina et al., 2014). The pPC9 plasmid is not self-transmissible but can be mobilized by other bacterial plasmids, a factor which increases the risk of the spread of drug resistance genes via horizontal gene transfer to new hosts including more pathogenically important species (Molina et al., 2014). In addition, Molina et al. (2014) showed that segments of the plasmid could have been acquired from human opportunistic pathogens or primary pathogens, such as *P. aeruginosa*, *Aeromonas hydrophila*, *Acinetobacter baumannii*, *Corynebacterium resistens* and *Enterobacteriaceae* (*Klebsiella pneumoniae*, *Salmonella enterica*). The presence of this exogenous DNA, including its high number antibiotic resistance cartridges, suggests a history of evolution in medical environments for HB3267.

### HB8234

Strain HB8234 revealed pathogenic potential on human skin tests, where it caused cytotoxicity and histological disorders; in addition, it further damaged the injured skin of Wistar rats, but it did not affect larvae survival. HB8234 was also sequenced recently by our research group (Molina et al., 2013) and it was found that the genome of this strain is significantly larger than other *P. putida* genomes (1.0 Mb larger), with most of the accessory DNA organized into 44 genetic islands, and having low or no homology to other *P. putida* genomes. This suggests that genome evolution in *P. putida* species is linked to the niche where these microbes develop.

### HB4184

This strain showed pathogenic traits, but only on human skin, where it caused a moderate cytotoxicity, histological damage and triggered an immune response. It did not cause damage on the skin of Wister Rats and did not affect insect survival. Taken together, these data reveal that HB4184 has a lower pathogenic potential than HB3267 or HB8234, however, its ability to form biofilms, which is superior to that of *P. aeruginosa* PAO1, is a worrisome trait. Nosocomial infections are often linked to devices surfaces that are colonized by biofilms (Abdallah et al., 2014); in fact, Carpenter et al. (2008) found that 93% of the reported cases of infections with *P. putida* were associated with the use of invasive medical devices. Bacterial biofilms are difficult to eradicate, since bacteria in biofilm exhibit higher resistance to antibiotic and disinfectant agents. Wang et al. (2010) found that urinary catheters were colonized by a mixed biofilm of *S. aureus*, *P. aeruginosa*, *Escherichia coli*, and *K. pneumoniae*. These mixed bacterial biofilms in medical environments represent the most favourable place for the exchange genetic material between clinical strains with the consequent risk of new pathogenic strains arising.

### HB13667

Strain HB13667 did not show pathogenic traits in any of the biological systems used in this study. HB13667 was associated with a strain of *Enterobacter cloacae* in the original clinical sample; it is possible that HB13667 was a merely coexisting and not associated with the clinical sequelae. In any case, the non-pathogenic character found for HB13667 is representative of most *P. putida* strains, but, as we have shown in this study, some dangerous traits can be acquired when the strains evolve in medical environments.

In short, our result show that *P. putida* isolated from clinical samples vary in their profile of antibiotic resistance and that, as for other microorganisms, horizontal gene transfer via plasmid is relevant in the clinical environment. Our data support the notion that the arsenal of accessory genes found in a clinical bacterial isolate is related to the environment in which those microbes develop and that strains of *P. putida* harbour a mosaic of genes often based on DNA exchange.

## Materials and Methods

### Bacterial Strains and Growth Conditions

Bacterial strains used in this work are listed in **Table 1**. *P. putida* clinical strains were isolated from clinic samples at Hospital Jean Minjoz (Besançon, France) and pre-identified as belonging to *P. putida*. *Pseudomonas* strains were routinely grown in Luria-Bertani (LB) medium at 30°C with shaking, unless other conditions are mentioned. *P. aeruginosa* PAO1 and *E. coli* were grown at 37°C.

### Identification and Typing of Strains using DNA Techniques

Chromosomal DNA was isolated using the Wizard Genomic DNA Purification Kit (Promega, USA). PCR amplifications were performed according to standard procedures using Euro Taq polymerase (EuroClone, Italy).

16S rDNA amplification was performed using the F8 and R798 primers (Liu et al., 2008). The REPc method allows the identification of *P. putida* strains (Aranda-Olmedo et al., 2002) since this primer amplifies only DNA from this species, and generates products of different size for each strain. Previously optimized PCR conditions were used (Aranda-Olmedo et al., 2002). Positive (*P. putida* KT2440) and negative (*E. coli*) DNA controls were included.

Multilocus sequence typing was carried out using a set of primers (listed in Supplementary Table S2) to amplify the complete sequence of five housekeeping genes: RNA polymerase sigma factor *rpoD* (1851 pb), DNA gyrase subunit B *gyrB* (2241 pb), *N*-(5′-phosphoribosyl) anthranilate isomerase *trpF* (621 pb), 6-phosphogluconate dehydratase *edd* (1827 pb), and recombinase A *recA* (1068 pb) as previously reported (Frapolli et al., 2007; Khan et al., 2008).

### Antibiotic Resistance Profile

Antibiograms were performed with commercial discs (Biomerieux); 31 different antibiotics were tested. Overnight bacterial cultures were spread on 240 mm × 240 mm LB plates, air dried in a laminar flow and then discs containing antibiotics were placed on the LB plates. Plates were incubated at 30°C for 16–18 h. Halos surrounding the discs were measured as an indication of inhibition of growth. Assays were repeated at least three times in duplicate.

### Semi-quantitative Determination of Biofilm Formation

Semi-quantitative determination of biofilm formation was performed as previously described (Christensen et al., 1985). Experiments were conducted in 24 well flat-bottomed polystyrene microtitre plates using M9 minimal medium supplemented with 0.2% glucose and 0.4% casamino acids in the presence of different *Pseudomonas* strains. Biofilm formation was quantified after 6 h by staining with crystal violet using the technique described by O’Toole and Kolter (1998). Two independent experiments were carried out for each strain and experiments run in quadruplicate.

### *Ex Vivo* Evaluation of the Different *P. putida* Strains on Human Skin

Live human skin was obtained from male patients subjected to circumcision surgery under local anesthesia. Once obtained, the skin was divided into 8 mm-diameter circular pieces using a biopsy punch, and kept submerged at 37°C in culture medium (DMEM with 10% fetal calf serum and antibiotics) for 2 h. Informed consent was obtained for the use of the skin in this study. This study was approved by the local research and ethics committee.

To determine the biological effects of each *P. putida* strain on cell viability of the human skin, 10^5^ cells of each strain were diluted in 20 μl of PBS and placed on the epithelial surface of each skin fragment. PBS without bacteria was used as negative control and 2% Triton X-100 detergent (Sigma, St. Louis, MO, USA) was used to induce cell death (positive control). In all cases, culture medium was added after 10 min and tissues were cultured at 37°C in a tissue incubator for 24 h.

To determine cell viability at the structural level, DNA and LDH quantification methods were used as previously described (Oliveira et al., 2014). Briefly, 10 μl of culture medium was diluted in nuclease-free distilled water to a final volume of 100 μl and free DNA was quantified using a SmartSpec^TM^ plus Spectrophotometer (BIO-RAD, Hercules, CA, USA) at 260–280 nm. For LDH quantification, 100 μl of culture medium was added to 100 μl of a commercially available immunological Cytotoxicity Detection Kit (Roche Applied Science, Mannheim, Germany), and the presence of the cytoplasmic enzyme was detected by colorimetric assay using an ELX-800 plate reader (Biotek, Winooski, VT, USA). Each sample was measured five times.

### *In Vivo* Evaluation of the Clinical *P. putida* Strains in Animal Models

The *in vivo* effects of the *P. putida* strains were evaluated on the skin of 12 Wistar laboratory rats. First, the dorsal skin of each animal was shaved and 10^5^ cells corresponding to each strain and control pathogenic bacteria (*P. aeruginosa* PAO1) and non-pathogenic bacteria (*P. putida* KT2440) were applied along with a non-bacterial control (PBS). To facilitate the entry of each bacterial strain into the rat skin, a freeze injury was generated at the site of the implant in half of the animals by using a metal rod submerged in liquid nitrogen for 30 s. All animals were evaluated and euthanized 24 h post-treatment for histological analysis.

### Histological Analysis and Immunohistochemistry

Human and rat skin samples evaluated in this work were fixed in 4% (v/v) formaldehyde, dehydrated in ethanol and embedded in paraffin for histological analysis. In all cases, 4 μm-thick sections were obtained and stained in hematoxylin and eosin and analyzed using a light microscope. For scanning electron microscopy, tissues were fixed in cacodylate-buffered 3% glutaraldehyde, dehydrated in increasing concentrations of acetone (30, 50, 70, 95, and 100%), critical-point dried, gold sputter-coated and analyzed in a scanning electron microscope (Quanta 200; FEI, Eindhoven, The Netherlands), using high vacuum mode.

Analysis of intercellular junctions was carried out by immunofluorescence with specific anti-zonula occludens 2 or anti-desmoplakin primary antibodies, and FITC-labeled secondary antibodies. Samples were analyzed using a confocal microscope. Evaluation of laminin, interleukin 1A (IL-1A) and 6 (IL-6), interferon (IFN) and tumor necrosis factor (TNF) was performed by immunohistochemistry. Briefly, tissue sections were deparaffinized, incubated in pH 6 citrate buffer for 40 min at 95°C for antigen retrieval, and primary antibodies were applied for 60 min at room temperature. Secondary antibodies were applied and the reaction was developed using a commercial 3-3′ diaminobenzidine kit (Vector Laboratories, Burlingame, CA, USA). Finally, samples were counterstained in Mayer’s hematoxylin and mounted on coverslips for light microscopy evaluation.

### Evaluation of the Clinical *P. putida* Strains using Insect Models

A stock colony of *C. carnea* (Stephens) has been maintained at the Estación Experimental del Zaidín since 2005 from larvae supplied by Koppert Spain (La Mojonera, Almería). On arrival larvae were individually transferred to petri dishes and reared on eggs of *Ephestia kuehniella* Zeller purchased from Biotop (Valbonne, France). After emergence, adults were placed inside plastic rearing boxes (approximately 100 specimens per box) with access to mineral water and an artificial diet consisting of 50% honey and 50% pollen spread over filter paper and sprinkled with quartz grains. The food and water were replenished weekly. The stock colony was maintained in a controlled environment cabinet at 25 ± 1°C, 50–60% relative humidity, and a photoperiod of 16:8 light:dark (L:D) h. New individuals were added monthly with an additional larvae supply.

*Chrysoperla carnea* larvae used in the experiments were obtained from eggs laid onto the removable lid of the rearing boxes. Over 300 eggs were collected, kept in individual Petri dishes (4.5 cm diameter), and allowed to develop. Larvae were supplied with *E. kuehniella* eggs *ad libitum* every other day. Eleven days after egg collection, 70 recently molted (less than 12 h) third instar larvae, of approximately the same length (4–6 mm) and weight (2–4 mg), were chosen for the experiment and allocated randomly (10 individuals) to each treatment. The larvae were then placed in pairs on solid LB agar on which 0.1 mlof a 10^8^ CFU/ml culture had been spread (five dishes per treatment) and kept in contact with the bacteria for an hour. The larvae were allowed to move freely on the agar surface but, whenever they climbed to the lid, this was tapped gently to make them fall, restricting their movement to the bottom assured maximum contact with the bacteria throughout the process. Once the inoculation process finished, the larvae were transferred individually to new Petri dishes, supplied with food, and kept again under standard larvae culture conditions. Larval and pupal mortality was assessed twice daily until emergence of the pharate adult. Pupal mortality inside the cocoon was recorded whenever the pupae started to blacken. The experiment used a randomized complete block design running the whole experiment up to six times (60 individuals per treatment).

Survival data was analysed using a frailty Cox proportional hazards model (Therneau and Grambsch, 2000). The blocking structure for experimental run was included in the model as a frailty term with a Gaussian error distribution. The frailty approach incorporates the frailty terms as random effects in the Cox proportional hazards model to control for the correlation in clustered survival data as is the case for each experimental run. The analysis was carried out using R software for windows (R Development Core Team, 2010) with functions from the packages survival and splines.

## Conflict of Interest Statement

The authors declare that the research was conducted in the absence of any commercial or financial relationships that could be construed as a potential conflict of interest.
