# Supplementary material for: Analysis of the pathogenic potential of nosocomial Pseudomonas putida strains
Source: Front Microbiol. 2015 Aug 25;6:871. doi: 10.3389/fmicb.2015.00871 (PMC4548156; doi:10.3389/fmicb.2015.00871)
Supplement: Supplementary file 1 [file Table_1.DOCX]

**Table S1**. Antibiogram assays using antibiotic disks .- Numbers indicate the size of the inhibition halo (cm) surrounding the antibiotic disc. Data are the average of 3 assays performed in duplicate with standard deviation below 5% of the given values

| **Antibiotic,**  **concentration (mg)** | **Strain** | | | | | | | | | **Antibiotic group** |
| --- | --- | --- | --- | --- | --- | --- | --- | --- | --- | --- |
|  | KT2440R | HB13667 | HB8234 | HB3304 | HB4184 | HB4477 | HB4557 | HB3536 | HB3267 |  |
| Ciprofloxacin, 5 | 2.8 | 0 | 0 | 0 | 2 | 0 | 0 | 0 | 0 | fluoroquinolone |
| Norfloxacin, 10 | 2.5 | 2.7 | 2.6 | 2.5 | 0 | 0 | 0 | 0 | 0 | fluoroquinolone |
| Pefloxacin, 5 | 1.9 | 1,8 | 0 | 0 | 0 | 0 | 0 | 0 | 0 | fluoroquinolone |
| Ofloxacin, 5 | 1.8 | 0 | 2.4 | 2.1 | 2.2 | 0 | 0 | 0 | 0 | fluoroquinolone |
| Nalidixic acid, 30 | 0 | 0 | 1.4 | 1.4 | 1.3 | 0 | 0 | 0 | 0 | quinolone |
| Erithromycin, 15 | 0 | 0 | 0 | 0 | 0 | 0 | 0 | 0 | 0 | macrolide a |
| Gentamycin, 10 | 1.8 | 1.6 | 0 | 1.7 | 1.5 | 0 | 0 | 0 | 0 | aminoglycoside |
| Kanamycin, 30 | 2 | 1.6 | 0 | 1.7 | 1.5 | 0 | 0 | 0 | 0 | aminoglycoside |
| Neomycin, 30 | 1.8 | 1.6 | 0 | 1.7 | 1.7 | 0 | 0 | 0 | 0 | aminoglycoside |
| Streptomycin, 10 | 0.9 | 1 | 0 | 0 | 0 | 0 | 0 | 0 | 0 | aminoglycoside |
| Amikacin, 30 | 0 | 1.7 | 2.1 | 1.9 | 1.7 | 1.9 | 2.1 | 1.9 | 1.7 | aminoglycoside |
| Netilmicin, 30 | 1.4 | 0 | 2.2 | 2.1 | 0 | 0 | 0 | 0 | 0 | aminoglycoside |
| Tetracycline, 30 | 0 | 1.2 | 1.6 | 1.6 | 1.6 | 0 | 0 | 0 | 0 | polyketide antibiotic |
| Polymyxin B, 300 | 1.3 | 0.8 | 0 | 0 | 0.9 | 0.9 | 1 | 1 | 1 | surfactant |
| Colistin, 50 | 1.5 | 0.8 | 0 | 1.2 | 0 | 1.8 | 1.1 | 1.2 | 1.2 | polymyxin |
| Trimethoprim, 20 | 0 | 0 | 0 | 0 | 0 | 0 | 0 | 0 | 0 | dihydrofolate reductase inhibitors |
| Chloramphenicol, 30 | 0 | 0 | 0 | 0 | 0 | 0 | 0 | 0 | 0 | bacteriostatic antimicrobial |
| Amoxycillin, 25 | 1 | 1 | 0 | 0 | 0.8 | 0 | 0 | 0 | 0 | ß-lactam antibiotic (penicillin) |
| Carbenicillin, 100 | 0 | 0 | 0 | 0 | 0 | 0 | 0 | 0 | 0 | ß-lactam antibiotic (penicillin) |
| Ticarcillin, 70 | 0 | 0 | 1.4 | 1.4 | 1.3 | 0 | 0 | 0 | 0 | ß-lactam antibiotic (penicillin) |
| Piperacillin, 10 | 0 | 0 | 1.9 | 1.9 | 1.7 | 0 | 0 | 0 | 0 | ß-lactam antibiotic (penicillin) |
| Ampicillin, 10 | 0 | 0 | 0 | 0 | 0 | 0 | 0 | 0 | 0 | ß-lactam antibiotic (penicillin) |
| Imipemen, 10 | 2.8 | 2.7 | 0 | 0 | 0 | 0 | 0 | 0 | 0 | ß-lactam antibiotic (carbapenem) |
| Cefotaxime, 30 | 1.5 | 1.3 | 0 | 0 | 0 | 0 | 0 | 0 | 0 | ß-lactam antibiotic (cephalosporin) |
| Ceftazidime, 30 | 1.6 | 1.4 | 2.3 | 2.3 | 1.9 | 0 | 0 | 0 | 0 | ß-lactam antibiotic (cephalosporin) |
| Ceftriaxone, 30 | 0 | 0 | 1.3 | 1.3 | 0 | 0 | 0 | 0 | 0 | ß-lactam antibiotic (cephalosporin) |
| Sulfonamide G, 20 | 0 | 0 | 0 | 0 | 0 | 0 | 0 | 0 | 0 | sulfonamides |
| Rifampicin, 30 | 0.6 | 1.2 | 1.7 | 2.2 | 1.4 | 1.9 | 1.8 | 1.9 | 1.6 | rifamycin group |
| Vancomycin, 30 | 0 | 0 | 0 | 0 | 0 | 0 | 0 | 0 | 0 | glycopeptide antibiotic |
| Esperamicin, 100 | 0 | 0 | 0 | 0 | 0 | 0 | 0 | 0 | 0 | chromoprotein enediyne antibiotics |
| Nitrofurantoin, 300 | 1.8 | 2.5 | 2.6 | 2.6 | 2.3 | 2.3 | 2.4 | 2.4 | 2.4 | nitrofurans |
