# Supplementary material for: Analysis of the pathogenic potential of nosocomial Pseudomonas putida strains
Source: Front Microbiol. 2015 Aug 25;6:871. doi: 10.3389/fmicb.2015.00871 (PMC4548156; doi:10.3389/fmicb.2015.00871)
Supplement: Supplementary file 2 [file Table_2.DOCX]

**Table S2**.- Oligonucleotide sequences used for Multilocus Sequence Typing.

| Primer | Sequence (5’-3’) | Reference |
| --- | --- | --- |
| edd1 | AGTCCAGTACATGCATCCGC | This study |
| edd2 | TCATTTGAGGTGCTCCAGGG | This study |
| edd3 | TCTGCGACAAGATCGTCCCC | This study |
| edd4 | ttgttggagatgccggaaacc | This study |
| edd5 | GGGTATCCAGCTGACCTGG | This study |
| edd6 | GCCGGCAGCCTGGAAGTGGTT | This study |
| edd7 | TTCCAGGGCCCGCGCTGCAA | This study |
| edd8 | GGGGATCTTGCCCGAGGCAC | This study |
| gltA1 | CCACATGGCTGACAAAAAAG | This study |
| gltA2 | TTACTTGCGGTCTTTCAGGG | This study |
| gltA3 | CCGCAACACCGCGAAATCTC | This study |
| gltA4 | GCGGGTACATCATCGGCTGG | This study |
| gltA5 | CCCGCGCGCCACCGTGATGAA | This study |
| gltA6 | GGGCGATCTCTTCCAGGCGC | This study |
| gyrB1 | ATGAGCGAAAATCAAACGTA | This study |
| gyrB10 | TTTCCACTTCAGGCAGCCATAC | This study |
| gyrB2 | TCAGAAGTCCAGGTTCGACA | This study |
| gyrB3 | CGCCCATGGCGGTTGTCGGT | This study |
| gyrB4 | TTCTTGAAGGTTTCAGCCGAT | This study |
| gyrB5 | GGCGACGACGCCCGTGAAGG | This study |
| gyrB6 | CCTCCGAGGAGACCAGCTTG | This study |
| gyrB7 | CACTGCGCTGGGCTGTGGCA | This study |
| gyrB8 | ACGGATGTGCGAACCGTCAACGTCA | This study |
| gyrB9 | CCAGGCGCGTCTGAACTCC | This study |
| ropD3 | CACCACCGAGGGTGGCCGCC | This study |
| ropD4 | GGGTCGATGTAACCGCTGAGA | This study |
| ropD5 | GACCAGACCTGGAGCGGTGA | This study |
| ropD6 | GGTCTCAAGGTCGATCAGCTT | This study |
| ropD7 | CAGGAAATGGGTCGCGAACC | This study |
| ropD8 | GGGGTTTCCATGGAGATCGG | This study |
| rpoD1 | ATGTCCGGAAAAGCGCAACA | This study |
| rpoD2 | TCACTCGTCGAGGAAGGAGC | This study |
| trpE1 | ATGAACCGCGAAGAATTCCT | This study |
| trpE2 | CCCAATCAATTTATCTGGCG | This study |
| trpE3 | CCGACGCGGTGGTGGTGTTC | This study |
| trpE4 | ttgcagacgtgcctggccctgtt | This study |
| trpE5 | GACCGAAGAGGCCGATCGTG | This study |
| trpE6 | ctcggtcaggcgcacactgcc | This study |
